# An animal model study of simplified optical navigation system-assisted percutaneous lumbar transforaminal puncture

**DOI:** 10.1186/s13018-025-06191-3

**Published:** 2025-08-12

**Authors:** B. N. Wenxiu Chai, Yuqing Chen, Li Sheng, Yong Huang

**Affiliations:** 1 Operating Room, The Affiliated Xinghua People’s Hospital, Medical School of Yangzhou University, Xinghua, Jiangsu China; 2 The Department of Orthopedics, The Affiliated Xinghua People’s Hospital, Medical School of Yangzhou University, Xinghua, Jiangsu China; 3 The Department of Neurosurgery, The Affiliated Xinghua People’s Hospital, Medical School of Yangzhou University, Xinghua, Jiangsu China

**Keywords:** Optical trackers, 3D slicer, Navigation, Puncture localization, Fluoroscopy

## Abstract

**Background:**

Optical navigation systems can provide real-time visualization of the positional relationship between surgical instruments and patient anatomy, making them suitable for assisting in puncture localization during percutaneous transforaminal endoscopic discectomy (PTED). However, current research on optically-navigated puncture localization during PTED is still limited, with high equipment costs and complex operational techniques being the main barriers to widespread adoption. 3D Slicer is a powerful open-source medical image analysis and visualization platform that can be integrated with external optical trackers to construct a low-cost optical navigation system for assisted puncture procedures. This study aims to develop a simplified optical navigation system based on an optical tracker and 3D Slicer software, and to evaluate its effectiveness in assisting percutaneous lumbar foraminal puncture through animal model experiments.

**Methods:**

A simplified optical navigation system based on an optical tracker and 3D Slicer software was developed. Eight fresh goat lumbar spine segments were randomly divided into the navigation group and control group. The two groups underwent the lumbar foraminal puncture procedure with different guidance modalities: the navigation group utilized the simplified optical navigation system, while the control group employed conventional C-arm fluoroscopic guidance. Each group contained 4 lumbar specimens, with percutaneous foraminal punctures performed bilaterally at the L2/3, L3/4, and L4/5 levels. Evaluation metrics(number of puncture attempts, fluoroscopy frequency and total puncture positioning time) were recorded and compared between groups.

**Results:**

All 48 lumbar transforaminal punctures (24 in each group) were successfully completed. The navigation group required significantly fewer puncture attempts (1.21 ± 0.51 vs. 5.13 ± 1.19) and fewer fluoroscopies (3.63 ± 0.77 vs. 12.88 ± 2.29) compared to the control group. However, the control group demonstrated shorter puncture positioning time (16.38 ± 2.58 min vs. 20.04 ± 1.68 min). The navigation group achieved a 79.17% first-attempt success rate. In the 5 unsuccessful initial attempts, fluoroscopy showed minimal deviation from the lumbar foramen, with all subsequent navigation-guided punctures being successful. The simplified optical navigation system required approximately 15 min for manual registration, demonstrated excellent stability without image lag or drift, and showed reliable performance with no system shutdowns or startup failures.

**Conclusion:**

The simplified optical navigation system developed using optical tracking technology and 3D Slicer software can effectively assist percutaneous lumbar transforaminal puncture procedures. This navigation system provides direct visualization of the target anatomy, enables precise localization of puncture points and accurate adjustment of needle trajectories, thereby reducing procedural uncertainty and technical difficulty. The system significantly improves puncture accuracy while markedly decreasing both the number of puncture attempts and fluoroscopy exposures compared to conventional techniques.

## Introduction

Safe and accurate puncture localization is a critical step in percutaneous transforaminal endoscopic discectomy (PTED) [1]. However, due to the narrow anatomical space of the lumbar foramen, accurate puncture localization in PTED remain technically challenging. Conventional fluoroscopy-guided freehand puncture techniques rely heavily on the surgeon’s experience and spatial orientation skills, often requiring repeated fluoroscopic imaging and multiple puncture attempts for successful placement. This steep learning curve can be intimidating for beginners, significantly hindering the widespread adoption of this technique [2, 3]. Optical navigation systems offer high precision and immunity to electromagnetic interference, making them suitable for assisting puncture localization in PTED. A few studies have explored the use of optical navigation for this purpose, demonstrating that it can reduce technical difficulty, improve puncture accuracy, and significantly decrease radiation exposure [4, 5]. However, the high cost of equipment and complex operational requirements have limited the widespread adoption of optical navigation systems. 3D Slicer, through the OpenIGTLink protocol, can interface with optical tracking devices to enable data transmission and reception, providing real-time navigation images for surgical guidance [6, 7]. In this study, we developed a low-cost simplified optical navigation system by integrating an optical tracker with 3D Slicer software. We conducted an animal model experiment to evaluate the feasibility and effectiveness of this simplified optical navigation system in assisting percutaneous lumbar foraminal puncture.

## Methods

### Materials and equipment

**Fig. 1:**
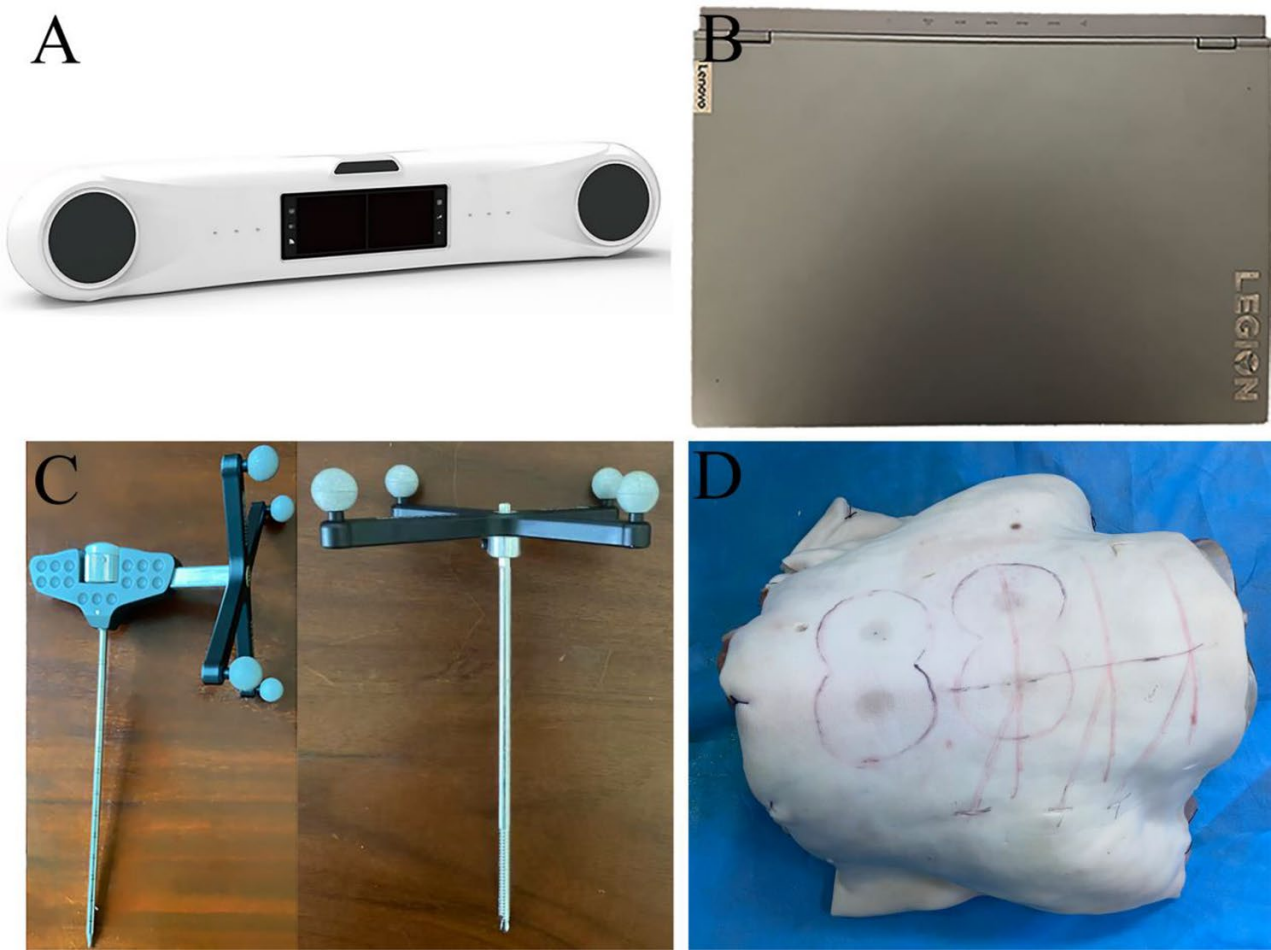
Materials and equipment. **A**: Near-infrared optical tracker. **B**: Laptop computer. **C**: Guided needle and reference frame with optical marker balls. **D**: Goat lumbar spine segment

**Fig. 2:**
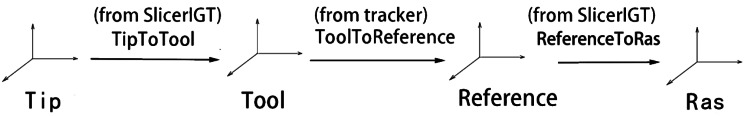
Principle of coordinate system construction for the optical navigation system integrating optical tracker and 3D Slicer software. The implementation of data transmission, surgical tool registration, and spatial registration between the optical tracker and 3D Slicer required three coordinate transformation matrices: TipToTool, ToolToReference and ReferenceToRas

**Fig. 3:**
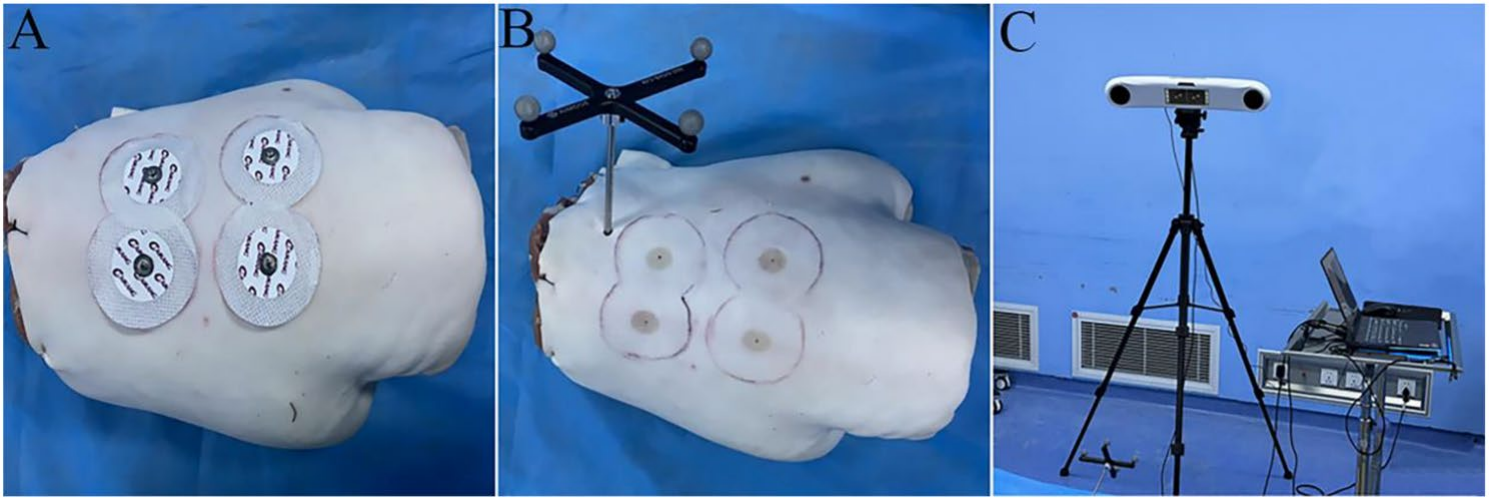
Preoperative CT scanning, reference frame installation and optical tracker adjustment. **A**: Preoperative CT scan of the goat lumbar spine in the prone position with four metal markers attached to the dorsal skin. **B**: Reference frame fixed to the spinous process of a cranial vertebra. **C**: System setup with adjustment of the optical tracker’s height, angle, and working distance (1 ~ 1.5 m)

**Fig. 4:**
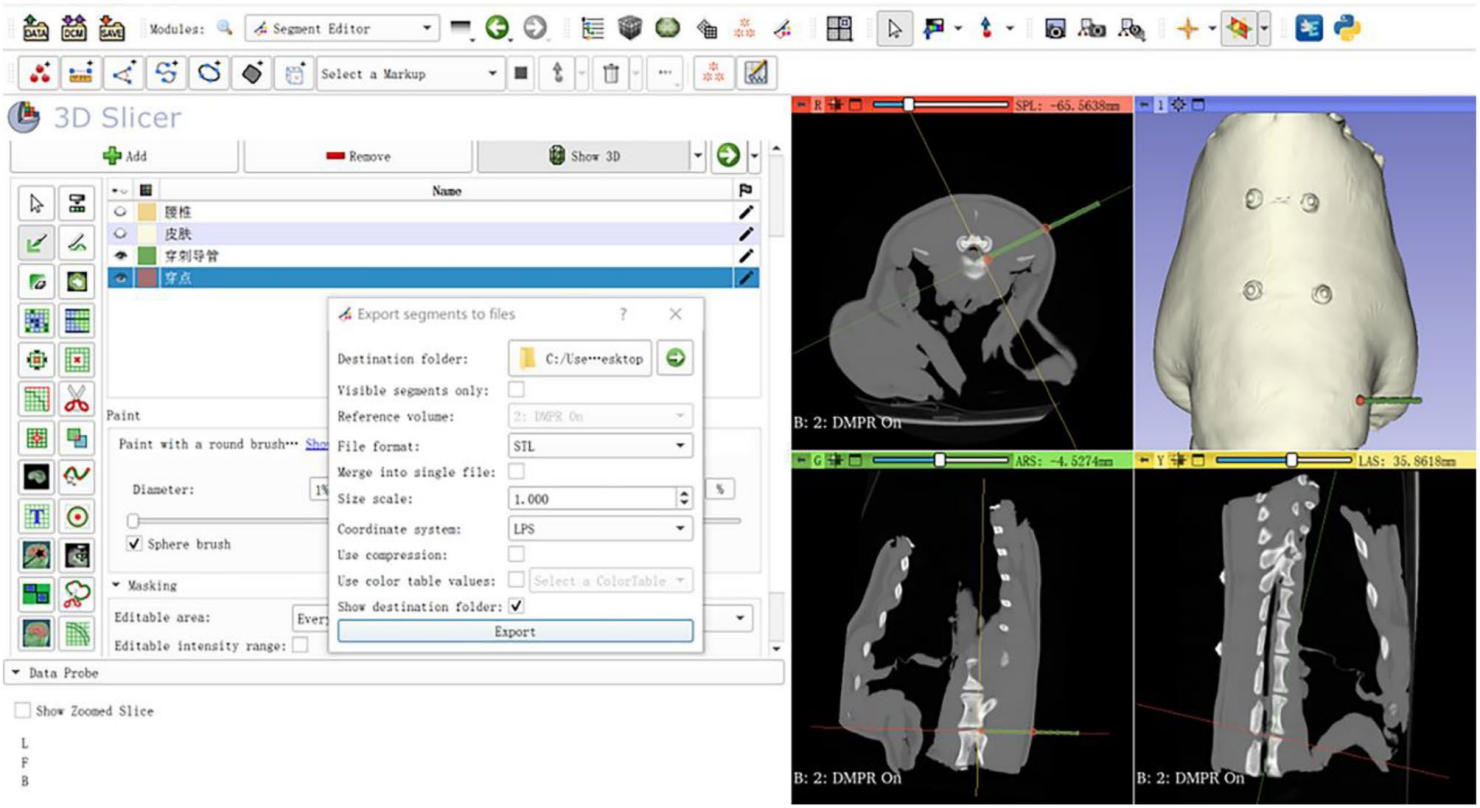
3D Slicer modeling. **A**: Reconstructed 3D models of the skin and lumbar vertebrae. **B**: Virtual puncture guide tube (50 mm length) generated along the planned trajectory. **C**: Spherical model (1 mm radius) marking the skin entry point

(1) A near-infrared optical tracking system (IME AP-STD-200): featuring binocular stereo vision technology with 0.12 mm tracking accuracy, 60 Hz sampling frequency, and 1–2.4 m working range, supporting data transmission via USB, Ethernet, or WiFi (Fig. 1A); (2) A high-performance Lenovo Legion laptop: AMD Ryzen 7 5800 H processor, 3.20 GHz) running Windows 10/11 OS (Fig. 1B); (3) Custom-designed guide needles with optical marker balls and reference frames (Fig. 1C); (4) Excised goat lumbar spine specimens (Fig. 1D).

### Development of a simplified optical navigation system for assisted puncture procedures based on optical tracker and 3D slicer software

The core technical challenge in developing the optical navigation system using the optical tracker and 3D Slicer software involved establishing data transmission, surgical tool registration, and spatial registration. To address this, we created a “ToolToReference” project file modified from the OpenIGTLink sample code available on the 3D Slicer official website. The system implementation required three coordinate transformation matrices: TipToTool, ToolToReference, and ReferenceToRas. In our study, “Tip” represented the guide needle’s tip, “Tool” denoted the guide needle itself, “Reference” indicated the goat lumbar spine, and “Ras” referred to the standard Right-Anterior-Superior coordinate system. The implementation process began with establishing data transmission between the optical tracker and 3D Slicer through the OpenIGTLinkIF module. With both the optically-marked guide needle and lumbar specimen positioned within the tracker’s field of view, executing the ToolToReference program generated and transmitted a 4 × 4 homogeneous matrix (representing the ToolToReference transformation) to 3D Slicer. The transformation matrix TipToTool, which relates the coordinate system of the needle tip to that of the fiducial marker balls on the guide needle, was obtained using the Pivot Calibration module in the IGT system, achieving guide needle registration. The transformation matrix ReferenceToRas, which converts the goat lumbar coordinate system to the RAS coordinate system, was acquired through the Fiducial Registration Wizard module in the IGT system, completing spatial registration. Ultimately, the coordinate systems of the guide needle, the goat lumbar spine, and the 3D lumbar spine images were unified (Fig. 2).

### Animal model experiment of percutaneous lumbar transforaminal puncture

Eight fresh goat lumbar spine segments (including skin, muscle, and bone tissues) were randomly divided into the navigation group and control group. The two groups underwent the lumbar foraminal puncture procedure with different guidance modalities: the navigation group utilized the simplified optical navigation system, while the control group employed conventional C-arm fluoroscopic guidance. Each group contained 4 lumbar specimens, with percutaneous foraminal punctures performed bilaterally at the L2/3, L3/4, and L4/5 levels. This yielded 24 puncture procedures per group (4 specimens × 3 levels × 2 sides). Evaluation metrics(number of puncture attempts, fluoroscopy frequency and total puncture positioning time) were recorded and compared between groups. These parameters were used to assess the efficacy of the simplified optical navigation system for lumbar foraminal puncture. The study protocol was approved by the institutional animal ethics committee of our hospital.

#### **Navigation-assisted puncture procedure** (Using a right transforaminal approach at the L3/4 level of a goat lumbar spine as an example)

**Fig. 5:**
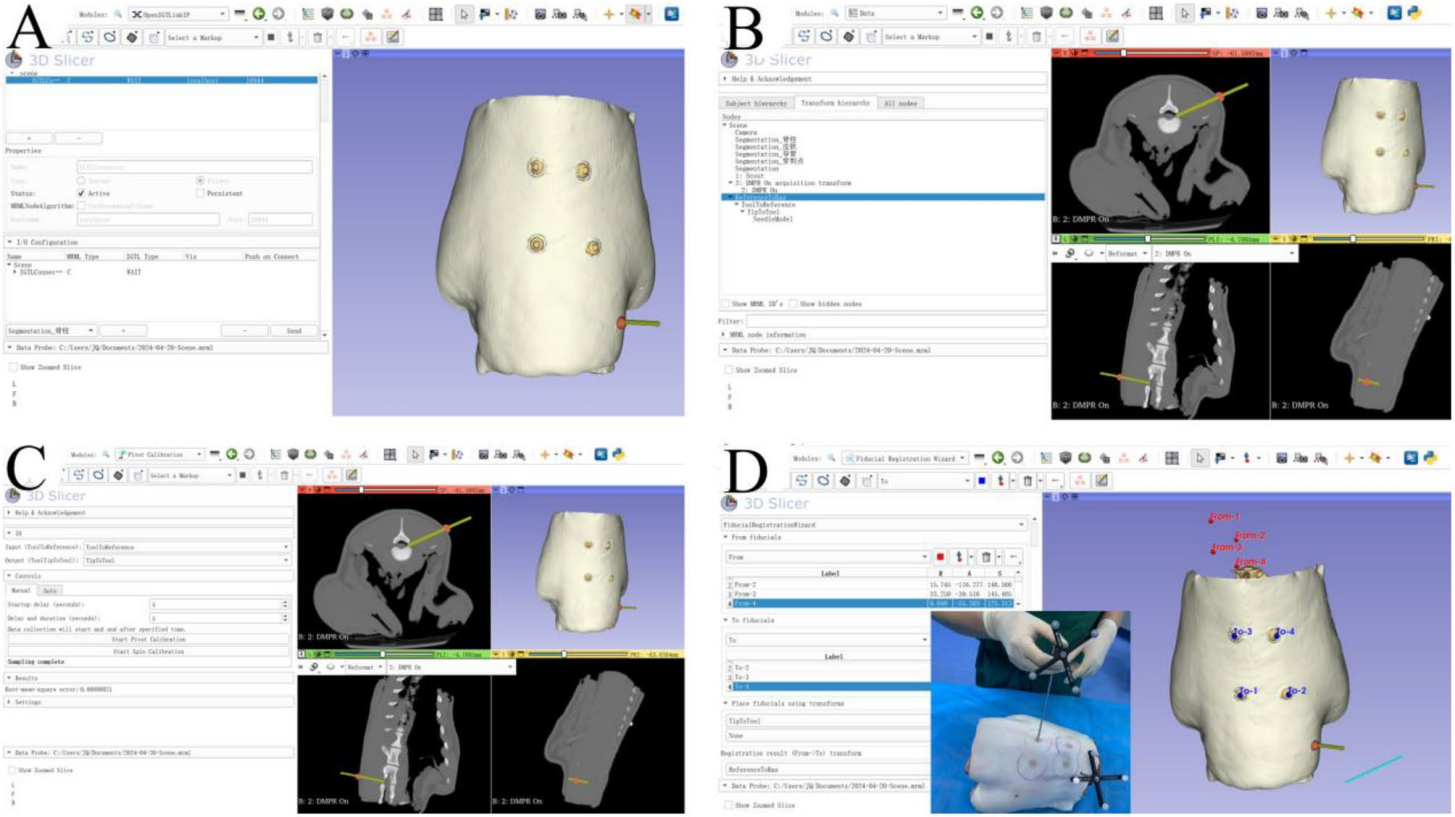
Navigation system registration process. **A**: OpenIGTLinkIF interface for real-time tracker-to-software data streaming. **B**: Virtual guide needle model with coordinate transformation matrix. **C**: Pivot calibration for needle tip registration (arrows indicate rotational movement). **D**: Fiducial-based spatial alignment using skin marker centroids

**Fig. 6:**
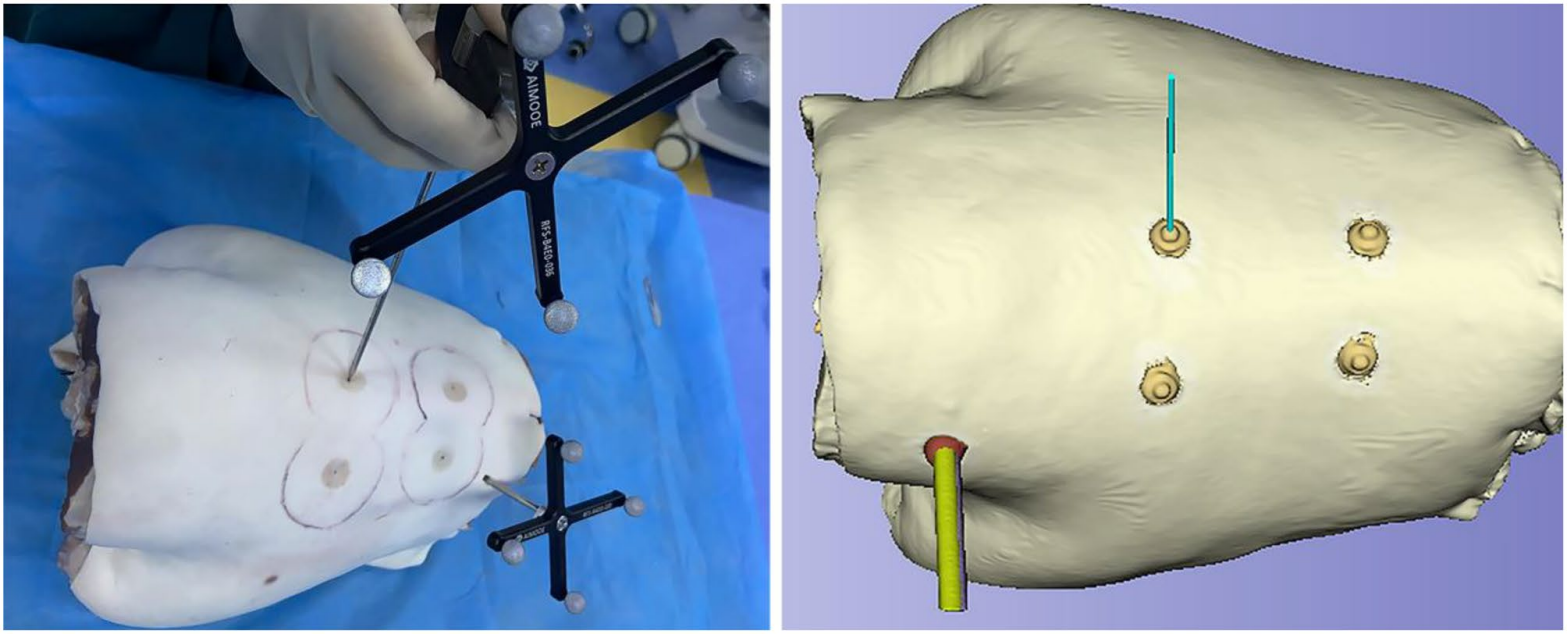
Demonstrates the preliminary accuracy assessment of the navigation system. The verification was performed by physically touching four pre-placed dorsal skin markers with the optically-tracked guide needle

**Fig. 7:**
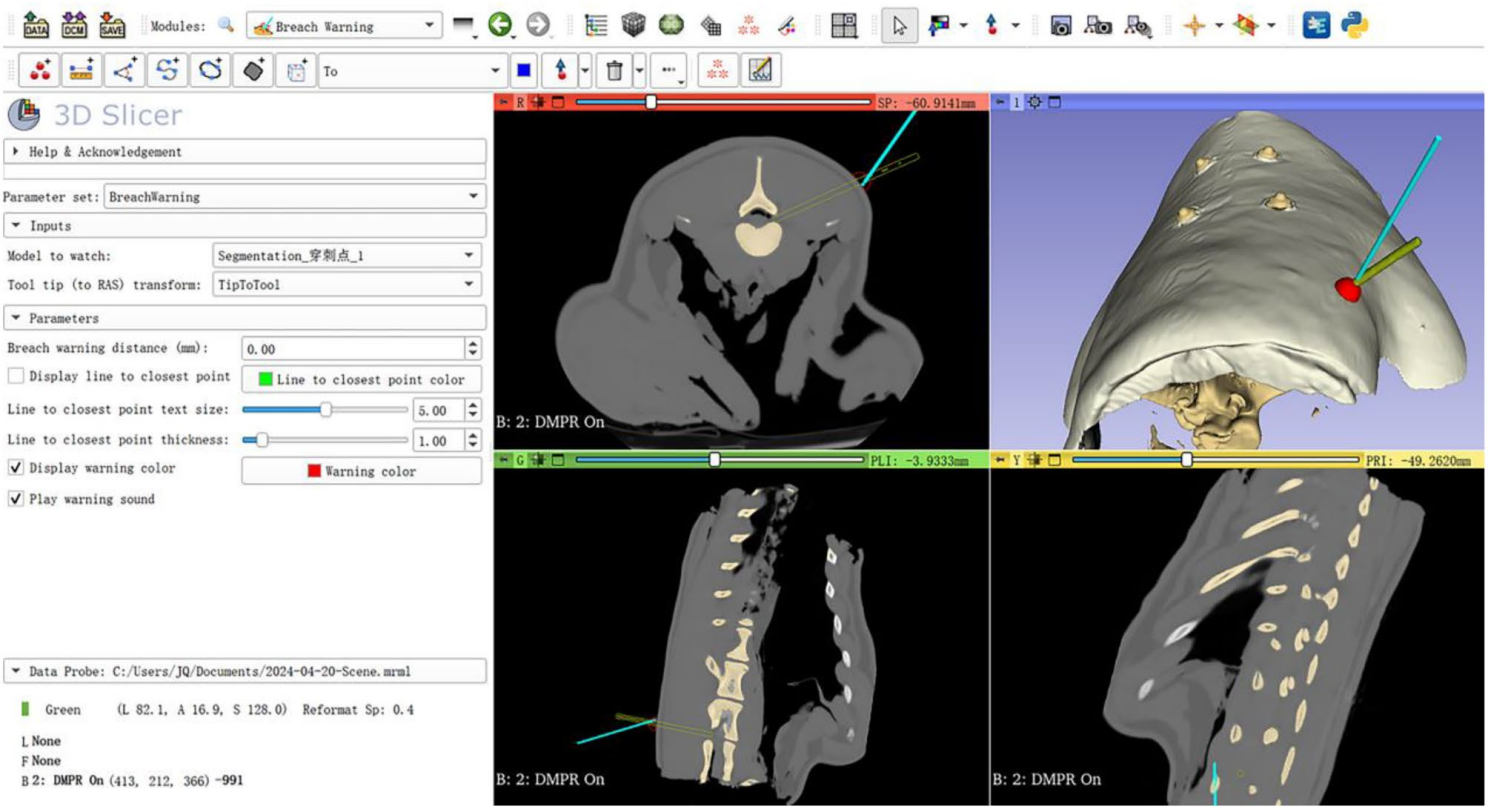
Precise localization of predetermined puncture points. The procedure combines real-time 3D image guidance through the 3D Slicer interface with the Breach Warning safety feature to achieve submillimeter targeting accuracy

**Fig. 8:**
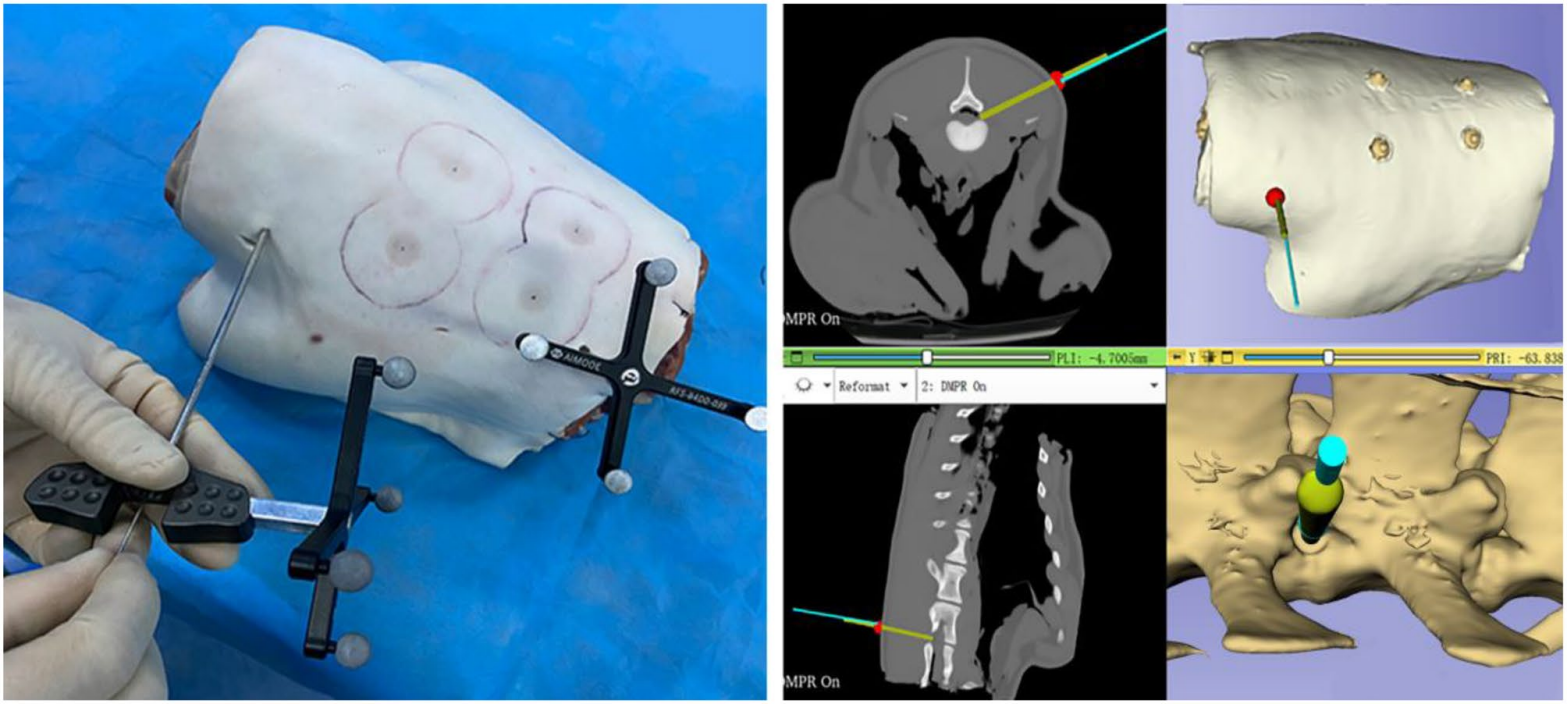
Real-time trajectory adjustment and instrument placement under navigation guidance. The navigation interface provided dynamic multi-planar visualization (axial, sagittal, and coronal) to precisely align the blue virtual guide needle with the green planned trajectory model in 3D Slicer. Through iterative adjustments, the operator achieved perfect spatial congruence between the navigation display and physical instrument. After trajectory confirmation, a 1.5 mm K-wire was advanced through the guide needle’s central channel under continuous navigation monitoring

**Fig. 9:**
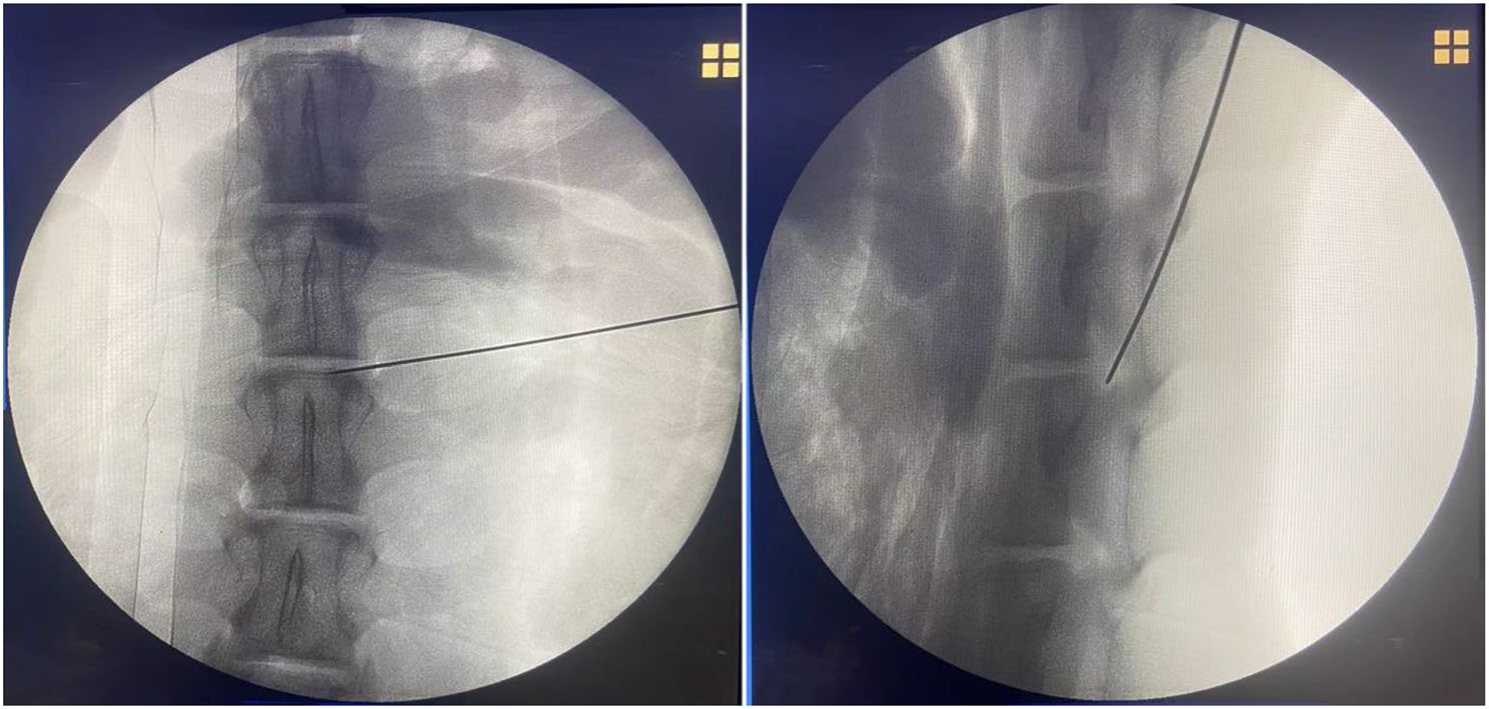
Fluoroscopic verification of K-wire position. Anteroposterior view: K-wire tip located between the medial pedicle border and spinous process midline. Lateral view: K-wire tip positioned at the posterior margin of the intervertebral disc space

(1) Preoperative CT scanning, reference frame installation, and optical tracker adjustment: Four metal markers were attached to the dorsal skin of the goat lumbar spine. A preoperative CT scan was performed with the specimen in the prone position (Fig. 3A). After removing the markers, their central points were marked as reference points for spatial registration. During the procedure, the goat lumbar spine was placed prone on the operating table. A hole was drilled into the spinous process of a cranial vertebra using an electric drill, followed by the installation of a reference frame (comprising a cross-shaped bracket with optical tracker marker balls and an external fixation screw). The reference frame was angled toward the optical tracker (Fig. 3B). A laptop was connected to the optical tracker, and the tracker’s height and angle were adjusted to ensure the entire goat lumbar spine remained within the field of view of its binocular cameras. The optical tracker was positioned 1 ~ 1.5 m away from the specimen (Fig. 3C).

(2) 3D Slicer Modeling: The DICOM-format CT images of the goat lumbar spine were imported into 3D Slicer for segmentation, generating 3D models of the skin and lumbar vertebrae. The puncture target was set at the right posterolateral region of the L3/4 intervertebral disc, while the entry point was located at the skin intersection of the posterior and lateral aspects at the L3/4 level. The planned needle trajectory was defined as the straight path connecting the entry point and the target. Using the “Draw Tube” function, a virtual puncture guide tube (extending 50 mm outward from the skin along the trajectory) was created. Additionally, a 1 mm-radius spherical model marking the entry point was generated using the “Sphere Brush” tool (Fig. 4).

(3) Data transmission, tool registration and spatial registration: First, the guide needle with optical marker balls and the goat lumbar spine equipped with a reference frame were individually placed within the optical tracker’s field of view. Tool identification files for both were created using AimTools software. The OpenIGTLinkIF module was then selected to establish a client connection, and upon running the TooltoReference program, a 4 × 4 homogeneous matrix was transmitted to 3D Slicer, achieving data transmission between the optical tracker and 3D Slicer (Fig. 5A). The Create Models module was used to generate a virtual guide needle model, while a four-level transformation matrix structure was established in the Data module (Fig. 5B). For tool registration, the Pivot Calibration module was employed: with the marker balls facing the optical tracker, the guide needle was rotated manually to determine the needle tip’s coordinate system based on the four marker balls at its proximal end (Fig. 5C). Finally, spatial registration was completed using the Fiducial Registration Wizard module, where the centers of four electrode patches served as fiducial points for point-based registration between the physical lumbar spine and its 3D image (Fig. 5D). This comprehensive process integrated the coordinate systems of the surgical tools, anatomical structures, and medical images for precise navigation.

(4) Navigation-guided puncture procedure: The navigation process commenced by verifying system accuracy through tactile confirmation of four skin fiducial markers using the guide needle (Fig. 6). The IGT module’s Breach Warning function was activated, establishing an alert sphere around the target puncture point. Under CT-based 3D image guidance, the guide needle was advanced toward the predefined entry point until the system triggered an automatic alert upon reaching the target (Fig. 7). Real-time trajectory adjustment was performed by aligning the blue virtual guide needle with the planned trajectory in multiplanar 3D Slicer views, with optional skin model transparency for direct foraminal visualization. After achieving optimal alignment, a 1.5 mm K-wire was advanced through the guide needle’s central channel, with immediate cessation upon encountering resistance (Fig. 8).

(5) Fluoroscopic Confirmation of K-wire Position: Final needle placement was verified under fluoroscopy, with successful positioning defined by: Anteroposterior view: K-wire tip located between the medial pedicle border and spinous process midline. Lateral view: K-wire tip positioned at the posterior margin of the intervertebral disc space (Fig. 9).

If the imaging results indicate that the puncture has failed, first assess the accuracy of the navigation, then perform the puncture again under navigation guidance. For each subsequent percutaneous lumbar transforaminal puncture, re-register the navigation before conducting the puncture under navigation guidance. Preoperative CT scanning, reference frame installation, positioning device adjustment, and 3D Slicer modeling are all part of the preoperative preparations. The puncture positioning time for the navigation group is measured from the start of navigation registration to the successful completion of the puncture.

#### Puncture procedure in the control group

**Fig. 10:**
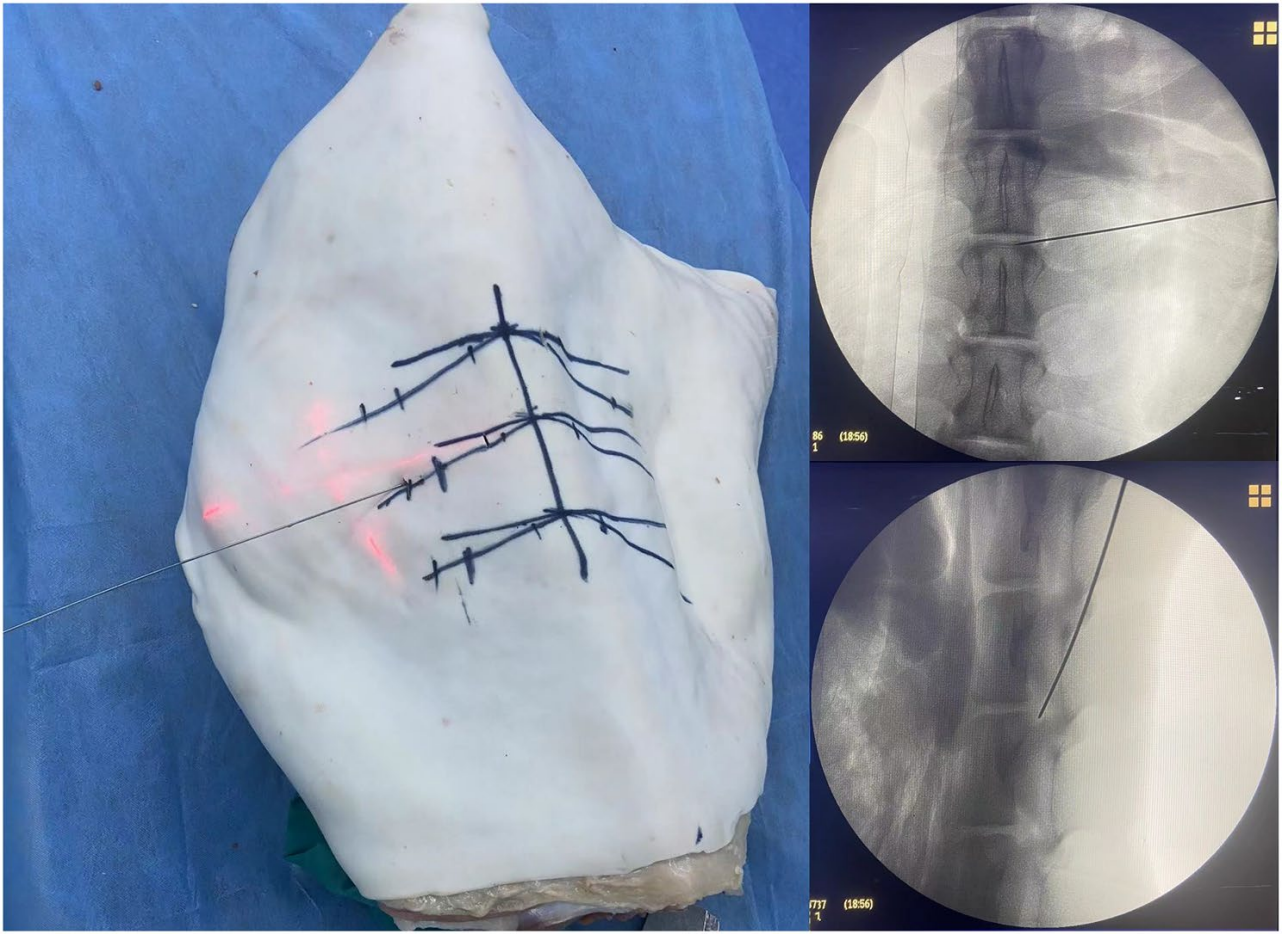
Puncture procedure in the control group. **A**: Demonstrates the standard fluoroscopic technique for lumbar transforaminal puncture. **B**: Confirms successful positioning through biplanar fluoroscopy

The goat lumbar spine was placed in a prone position on the operating table. Under anteroposterior (AP) fluoroscopy, puncture points were sequentially marked on both sides of the L2/L3, L3/L4, and L4/L5 segments. The puncture points were located along the line connecting the midpoint of the lower vertebral endplate and the tip of the superior articular process, approximately 6 ~ 8 cm lateral to the midline (at the junction of the posterior and lateral skin). A 16G puncture needle was used to perform the lumbar transforaminal approach puncture under conventional fluoroscopy guidance. The puncture direction was repeatedly adjusted under continuous fluoroscopy until successful puncture was achieved. A successful puncture was confirmed when the needle tip was positioned between the spinous process midline and the medial edge of the pedicle on AP fluoroscopy, and at the posterior edge of the intervertebral disc on lateral fluoroscopy (Fig. 10).

### Statistical methods

Statistical analysis was performed using SPSS 25.0 software. Categorical data were expressed as counts and compared using Pearson’s chi-square test or Fisher’s exact test. Continuous data were expressed as mean ± standard deviation. Normality tests were conducted for all continuous data. For data conforming to a normal distribution, the independent samples t-test was used for comparison between groups. For data not conforming to a normal distribution, the Mann-Whitney U test was applied. A p-value of < 0.05 was considered statistically significant.

## Results

All 48 lumbar transforaminal punctures (24 in each group) were successfully completed. The navigation group required significantly fewer puncture attempts (1.21 ± 0.51 vs. 5.13 ± 1.19) and fewer fluoroscopies (3.63 ± 0.77 vs. 12.88 ± 2.29) compared to the control group. However, the control group demonstrated shorter puncture positioning times (16.38 ± 2.58 min vs. 20.04 ± 1.68 min)(Table 1). The navigation group achieved a 79.17% first-attempt success rate. In the 5 unsuccessful initial attempts, fluoroscopy showed minimal deviation from the lumbar foramen, with all subsequent navigation-guided punctures being successful. The simplified optical navigation system required approximately 15 min for manual registration (from tool recognition file creation to point-matching completion), demonstrated excellent stability without image lag or drift, and showed reliable performance with no system shutdowns or startup failures.

**Table 1:** Comparison of operative parameters between the two groups

Parameter	Navigation group (*n* = 24)	Control group (*n* = 24)	*P* value
Number of puncture attempts	1.21 ± 0.51	5.13 ± 1.19	< 0.05*
Fluoroscopy frequency (times)	3.63 ± 0.77	12.88 ± 2.29	< 0.05*
Puncture positioning time (min)	20.04 ± 1.68	16.38 ± 2.58	< 0.05*

Note: Values are presented as mean ± SD or number (%). *Mann-Whitney U test

## Discussion

This study developed a low-cost optical navigation system by integrating the optical tracker with open-source 3D Slicer software for percutaneous lumbar transforaminal puncture procedures in goat models. Comparative experiments between this simplified optical navigation system and conventional fluoroscopy-guided techniques demonstrated that although the navigation system required more preoperative preparation, it could clearly define puncture trajectories and reduce procedural blindness. Compared to conventional fluoroscopy-assisted techniques, the optical navigation system significantly reduced puncture difficulty, improved accuracy, and markedly decreased both the number of puncture attempts (1.21 ± 0.51 vs. 5.13 ± 1.19) and fluoroscopy exposures (3.63 ± 0.77 vs. 12.88 ± 2.29). These animal model experiments confirm that the navigation system constructed with optical tracker and 3D Slicer software can effectively assist percutaneous lumbar transforaminal puncture, offering a potentially valuable low-cost solution for precision surgical punctures.

The Aimooe optical tracker exhibits technical characteristics including submillimeter accuracy, low latency, and multi-marker tracking capability, making it particularly suitable for medical optical navigation applications. The system requires a laptop with robust processing power, large memory capacity, and high-performance graphics capabilities to ensure optimal image processing and visualization performance. Lower-specification computers may experience system lag or unexpected shutdowns. 3D Slicer serves as a powerful open-source platform for medical image analysis and visualization, capable of rapidly reconstructing 3D models of various anatomical structures and precisely planning puncture trajectories [8]. Through the OpenIGTLink protocol, it can connect with optical trackers for bidirectional data transmission, providing navigation images for the system, making it a free yet powerful solution for puncture navigation [9–11]. The key technical challenges in building this navigation system involve establishing data transmission, tool registration, and spatial registration between the optical tracker and 3D Slicer. Sample implementations are available on the 3D Slicer website, where the OpenIGTLink module under the IGT module handles data transmission, the Pivot Calibration module accomplishes surgical tool registration, and the Fiducial Registration Wizard module performs point-matching spatial registration - all featuring straightforward operational procedures.

As a preoperative CT-based 3D image navigation system, this simplified optical navigation shares the inherent limitations of requiring manual registration and lacking intraoperative CT image updates. For successful percutaneous lumbar transforaminal puncture assistance, maintenance of consistent positioning between preoperative CT scanning and the actual procedure is crucial [12 ~ 15]. Significant positional changes would render preoperative CT images inaccurate for reflecting real-time lumbar anatomy [16], and could also alter the spatial relationship between skin markers and vertebral structures, potentially introducing substantial registration errors when using surface markers as reference points. In this study using goat lumbar specimens, near-perfect position consistency was maintained between scanning and procedures, with four clearly identifiable skin electrodes serving as registration reference points to minimize selection and manual operation errors. Experimental results showed a 79.17% first-attempt success rate for the navigation system in assisting transforaminal puncture, with no significant deviations observed even in unsuccessful attempts. The system’s excellent precision primarily benefits from the Aimooe tracker’s inherent high registration accuracy (0.12 mm) and robust data transmission with 3D Slicer. The observed application errors remained within acceptable ranges for lumbar transforaminal puncture assistance. This navigation system shows potential for assisting various precision surgical punctures, particularly in rigid anatomical structures like the cranium and bones, including applications such as hematoma drainage, trigeminal ganglion puncture, pedicle screw placement, pelvic fracture screw fixation, and sacroiliac screw insertion. Previous studies have successfully implemented similar optical navigation systems for abdominal procedures with satisfactory clinical outcomes [6, 7].

The navigation system requires rigid guide needles where the spatial relationship between optical marker balls at the needle base and the needle tip must remain constant, necessitating larger-diameter instruments. However, typical lumbar transforaminal puncture needles (1 ~ 2 mm diameter) are prone to deformation during use. Directly attaching optical markers to these slender needles for navigated procedures would likely lead to failure due to bending. Our solution employs larger guide needles as “external puncture conduits” to establish entry points and trajectories under navigation guidance, through which K-wires are then advanced. While the navigation cannot track the K-wire’s real-time position, intermittent minimal fluoroscopy can confirm placement. Electromagnetic navigation systems can track flexible instruments, with some studies reporting successful application in transforaminal puncture with reduced procedure times [17, 18], though magnetic interference from various operating room equipment remains a significant limitation hindering clinical adoption.

The simplified optical navigation system involves complex preparatory workflows including preoperative CT scanning, trajectory planning, 3D modeling, followed by intraoperative reference frame placement, manual tool registration, and spatial registration - each step requiring meticulous execution to ensure accuracy. Using guide needles as external conduits means the system cannot display real-time K-wire positions, with potential for trajectory deviation if wire deformation occurs. Therefore, K-wires should have adequate diameter and be advanced carefully to minimize bending. To avoid errors, users should first become proficient in 3D Slicer’s relevant modules and grasp the fundamentals of optical navigation. Given its simple design and workflow, the simplified optical navigation system offers a short learning curve.

These animal model experiments preliminarily demonstrate that the simplified optical navigation system can effectively assist lumbar transforaminal puncture. Despite requiring substantial preparation, the system provides direct visualization of targets and precise trajectory adjustment, significantly reducing both puncture attempts and radiation exposure. Due to the time-consuming system preparation and trajectory adjustment procedures of the simplified optical navigation system, the positioning time of the navigation group is significantly longer than that of the conventional puncture group. Although the time required for system preparation and trajectory adjustment can be gradually reduced as operators gain experience, the potential time savings remain limited. In the future, we can further optimize the relevant modules of 3D Slicer to streamline the workflow of this navigation system, thereby shortening the system preparation time. The anatomical structure of goat lumbar vertebrae differs from that of human lumbar vertebrae (including vertebral body size, soft tissue density, and anatomical landmarks). These anatomical differences may influence the performance of the navigation system in clinical applications. Substantial disparities between controlled animal experiments and clinical environments - factors that may affect generalizability and reliability.

## Conclusion

The animal model study demonstrates that the simplified optical navigation system developed using optical tracking technology and 3D Slicer software can effectively assist percutaneous lumbar transforaminal puncture procedures. This navigation system provides direct visualization of the target anatomy, enables precise localization of puncture points and accurate adjustment of needle trajectories, thereby reducing procedural uncertainty and technical difficulty. The system significantly improves puncture accuracy while markedly decreasing both the number of puncture attempts and fluoroscopy exposures compared to conventional techniques. This research presents a cost-effective optical navigation solution with satisfactory accuracy for surgical puncture procedures.

## Data Availability

No datasets were generated or analysed during the current study.
